# The Role of English Proficiency in HPV and HPV Vaccine Awareness: A Cross-Sectional Study Across Race/Ethnicity

**DOI:** 10.31557/APJCP.2021.22.2.349

**Published:** 2021-02

**Authors:** Hee Yun Lee, Yan Luo, Jessica Neese, Casey Daniel, Hyeouk Chris Hahm

**Affiliations:** 1 *School of Social Work, The University of Alabama, Tuscaloosa, AL, USA. *; 2 *USA Mitchell Cancer Institute, University of South Alabama, Mobile, AL, USA. *; 3 *School of Social Work, Boston University, USA.*

**Keywords:** Human papillomavirus (HPV) awareness, human papillomavirus (HPV) vaccine awareness

## Abstract

**Objective:**

Human papillomavirus (HPV) is the most common sexually transmitted infection in the United States and associated with over 90% of cervical cancer cases. The present study aimed to examine levels of HPV and HPV vaccine awareness and associated factors, particularly English proficiency, across various racial/ethnic groups.

**Methods:**

Two cross-sectional datasets, 2017 and 2018 Health Information National Trends Survey (HINTs), were analyzed for the current study. Binary logistic regression analyses were conducted.

**Findings:**

Non-Hispanic Whites had the highest levels while non-Hispanic Asians had the lowest levels of awareness regarding HPV and the HPV vaccine. English proficiency was significantly associated with increased HPV awareness among all groups except non-Hispanic Asian, and was significantly associated with greater HPV vaccine awareness in all groups.

**Discussion:**

In order to promote health equity across race/ethnicity, language barriers among minorities should be addressed when implementing health education on HPV and the HPV vaccine.

## Introduction

With an estimated 14 million new cases each year, human papillomavirus (HPV) is the most common sexually transmitted infection (STI) in the United States (U.S.) (Centers for Disease Control Prevention, 2018). Additionally, HPV is associated with 90% of anal, 60% of penile, 70% of vaginal and vulvar, 71% of oropharyngeal, and over 90% of cervical cancers (Centers for Disease Control Prevention, 2017; National Cancer Institute, n.d.). Fortunately, HPV infection can be prevented through the administration of a multi-dose vaccine, which was approved by the U.S. Food and Drug Administration (FDA) in 2006 (U.S. Food and Drug Administration (FDA), 2009; National Cancer Institute, n.d.). While recommended by the U.S. Advisory Committee on Immunization Practices (ACIP) for male and female adolescents ages 11-12, the HPV vaccine can be initiated as early as age 9 with catch-up doses recommended up to age 26 (Centers for Disease Control Prevention, 2017; Meites et al., 2019; National Cancer Institute, n.d.). In addition, shared clinical decision-making regarding potential HPV vaccination for adults between 27 and 45 years old was recommended by ACIP (Meites et al., 2019). Although this safe, effective vaccine is widely available, rates of HPV vaccination uptake and completion have remained far below the Healthy People 2020 goal of 80% coverage (Healthy People 2020, n.d.). National Immunization Survey-Teen (NIS-Teen) data demonstrated that national coverage with ≥1 dose of HPV vaccine was 68.1% among males and females ages 13-17 and that the percentage of these adolescents who were up-to-date with HPV vaccination was 51.1% (Centers for Disease Control Prevention, 2017). Despite increases in HPV vaccination awareness and uptake since its introduction in the U.S., there are substantial HPV vaccine disparities among minorities who also experience a disproportionately high rate of HPV-related cancers (Daley et al., 2011; Gelman et al., 2011). 

Several demographic factors have been examined to determine their potential associations with HPV and HPV vaccine awareness. With respect to gender, HPV and HPV vaccine awareness levels have remained consistently higher among women compared with their male counterparts (Blake et al., 2015; Boakye et al., 2017; Wheldon et al., 2019). By analyzing the data with 6,862 American adult respondents, Boakye et al., (2017) found that 74.5% of female participants had heard of HPV and 76.4% of females had heard of the HPV vaccine, as compared to 57.5% of males had heard of HPV and 55.2% of males had heard of the HPV vaccine. Another study conducted by McBride and Singh (2018) to examine awareness of HPV and the HPV vaccine among 3,103 American adults, reported a higher level of HPV awareness among females than males (59.72% versus 43.8%). Findings were in line with Boakye et al., (2017)’ study; however, findings indicated that males had higher HPV vaccine awareness (47.28%) than females (25.61%) (McBride and Singh, 2018). Interestingly, male participants of the McBride and Singh (2018) study were statistically more likely to have heard of the HPV vaccine, but this finding has not been replicated in other studies (Gelman et al., 2011; Blake et al., 2015). Age also appears to be associated with awareness, Blake and colleagues (2015) found that younger participants tended to have higher levels of HPV and HPV vaccine awareness than older participants. Other demographic factors associated with HPV and HPV vaccine awareness include rural/urban residence, education, marital status, annual income, children in household, and health insurance status (Gelman et al., 2011; Blake et al., 2015; McBride and Singh, 2018; Mohammed et al., 2018).

Race/ethnicity may be among the most significant factors influencing HPV awareness and HPV vaccination decision making. In the U.S., non-Hispanic White, regardless of gender, consistently indicate higher levels of awareness than other races/ethnicities such as African Americans and Hispanics (Boakye et al., 2017; Ojinnaka et al., 2017). Study findings from McBride et al., (2018) and Blake et al., (2015) determined that race/ethnicity other than non-Hispanic White was negatively associated with HPV and HPV vaccine awareness. Similarly, Gelman et al., (2011) found that, compared to 89% of non-Hispanic White participants, only 62% of Hispanic participants, 75% of African American participants, and 69% of participants from other races such as Asian Americans had heard of the HPV vaccine. Although frequently combined into broader categories such as “other races,” Asian Americans appear to have the lowest level of HPV awareness of all major racial/ethnic groups (Garcini et al., 2015). Another study indicated that only 59% of Asian/Pacific Islander parents were aware of the HPV vaccine compared to 90% of White, 65% of Latino, and 74% of African American (Nonzee et al., 2018). 

One variable that may serve as an extension of race/ethnicity is Limited English Proficiency (LEP). According to a report from American Community Survey in 2012, only half of the foreign-born population spoke English “very well” or better, while 21% of the foreign-born population spoke English “well”, whereas 19% spoke English “not well” and 10% said they did not speak English at all (Gambino et al., 2014). The reports also showed that the foreign-born population from Asia had more limited English proficiency compared to those from Africa, Europe, Europe, or Australia (Gambino et al., 2014). Moreover, the foreign-born population from Latin America and the Caribbean had the most limited English proficiency (Gambino et al., 2014). LEP has demonstrated a negative association with HPV vaccine uptake among Vietnamese American women (Jenny et al., 2013) and Chinese American women (Nguyen et al., 2012), and with HPV literacy among Korean Americans (Lee et al., 2018). Additionally, Hispanic parents have identified LEP as a barrier to the HPV vaccine (Luque et al., 2012). While the aforementioned studies demonstrated the relationship between LEP and the HPV vaccine, only Nguyen et al., (2012) reported the relationship between LEP and HPV awareness. This study suggested that low levels of HPV awareness (19%) among Chinese American female participants were significantly associated with LEP. Although previous studies have investigated the role of LEP in HPV prevention, the majority have targeted Asian females, and only one among Hispanic females. In addition, previous studies have only examined associations between LEP and HPV vaccine receipt, rather than awareness of HPV and the HPV vaccine. There is a paucity of literature examining the role of LEP in improving awareness of HPV and the HPV vaccine, especially among racial/ethnic groups are inadequate. To fill this gap, the current study aims to investigate the levels of HPV and HPV awareness and associated factors, particularly looking at the role of English proficiency among four racial/ethnic groups in the U.S.: non-Hispanic Whites, non-Hispanic African Americans, Hispanics to be consistent with non-Hispanic Whites, non-Hispanic African Americans, non-Hispanic Asians.

## Materials and Methods

*Research Method and Data Source*

The current study utilizes a cross-sectional research design. We analyzed two cross-sectional datasets from the National Cancer Institute’s (NCI) Health Information National Trends Survey (HINTS). The two datasets were merged (HINTS 5, Cycle 1, and Cycle 2, Total N = 5,868) to increase each racial/ethnic group’s sample size. These HINTS data were collected through self-administrated mailed surveys between January 2017 and May 2018 to learn about the American’s public use of information related to cancer. By applying a two-stage sampling strategy, a stratified sample of addresses was selected from a file of residential addresses, followed by a selection of one adult within each sample household. Under this sampling strategy, the samples in this study were considered representative of the national population; therefore, sample weight was not applied in this study. Additional information regarding data collection for HINTS 5, Cycle 1 and Cycle 2 is reported in its methodology reports (National Cancer Institute, 2017, 2018). To achieve the current study’s research aims, this study included four racial/ethnic groups: non-Hispanic Whites, non-Hispanic African Americans, Hispanic, and non-Hispanic Asians. 

*Dependent Variables*

HPV and HPV vaccine awareness were measured as two dependent variables. HPV awareness was measured by asking the respondents in HINTS 5: Have you ever heard of HPV? HPV stands for Human Papillomavirus. It is not HIV, HSV, or herpes (0=no, 1=yes). The HPV vaccine awareness was measured by using the following question in HINTS: A vaccine to prevent HPV infection is available and is called the HPV shot, cervical cancer vaccine, GARDASIL^®^, or Cervarix^®^. Before today, have you ever heard of the cervical cancer vaccine or HPV shot? (0=no, 1=yes). 

*Independent Variable*

English proficiency in HINTS was measured from the question, “How well do you speak English?” using a 4-point Likert scale, which was dichotomized into (0=well/not well/not at all, 1=very well). English proficiency was categorized in such way (speak English very well versus well/not well/not at all) to determine the overall proficiency impact. Individuals who report speaking English very well are presumed to be native English speakers or to have lived in the U.S. longer than those who report speaking English well/not well/not at all. Examining this nuanced distinction in proficiency will further elucidate the extent to which HPV and HPV vaccine awareness are impacted by English proficiency and identify groups in need of targeted interventions. 

*Covariates*

Sociodemographic characteristics (i.e., age, gender, race/ethnicity, marital status, educational attainment, and household income), health care resources (i.e., regular health care provider, frequency of visiting health care providers, and health insurance coverage), caregiver of children, number of children in the household, and self-reported health status were included in the analysis as covariates. Age was included as a continuous variable, while the remaining were classified into dichotomous variables: gender (0=male, 1=female), marital status (0=never married or other, 1=married or living as married), educational attainment (0=less than bachelor’s degree, 1=bachelor’s degree or higher), regular health care provider (0=no, 1=yes), health insurance coverage (0=no, 1=yes), caregiver of children (0=no, 1=yes), and health status (0=poor or fair, 1=excellent or very good or good). Household income was categorized into three groups (0=$0-$19,999, 1=$20,000-$74,999, 3=more than $75,000) as well as the frequency of visiting health care providers (0=0 time, 1=1-3 times, 2=more than 4 times) and the number of children in the household (0=0, 1=1-2, 2=more than 3). 

*Statistical Analysis*

Univariate analysis was used to examine descriptive information for each variable. Separate bivariate analyses were applied to examine the correlation of each variable with HPV awareness and HPV vaccine awareness. Crosstabs were generated for categorical variables, and an independent sample t-test was used for the only continuous variable, age. Lastly, binary logistic regression analyses across race/ethnicity were conducted on all variables to predict their association with HPV awareness and HPV vaccine awareness. All statistical analyses were performed using SPSS 24.0. (IBM Corp., 2013).

## Results

*Sociodemographics of the Sample*

Table 1 shows the study sample’s overall sociodemographic characteristics. Almost two-thirds of participants were non-Hispanic White, 14.5% were non-Hispanic African American, 15.1% were Hispanic, and 4.6% were non-Hispanic Asian. Participants tended to be older (Mean=55.8, SD=16.40), more than half were female (55.7%), married or living as married (54.2%), and did not have a bachelor’s degree (53.7%). Regarding income, 15.8% reported annual household income less than $19,999, 41.3% reported between $20,000 and $74,999, and 34.5% reported more than $75,000. Approximately 70% of participants had a regular health care provider. Small percentages of participants did not have health insurance coverage (4.0%) or cared for a child (6.3%). Although 14.3% reported visiting healthcare providers 0 times in the past 12 months, half of the participants (50.2%) reported 1 to 3 visits, and 34.6% reported 4 or more visits in the past year. Furthermore, 71.5% of the respondents did not have a child in the household, while 19.5% had 1 or 2 children, and 4.6% had more than 3 children. Most participants stated their health status as good/very good/excellent (83.5%) and reported themselves to speak English very well (87.9%). 

*HPV Awareness and the HPV Vaccine Awareness*

Bivariate analysis showed a significant difference in all variables among participants who had heard of HPV and among participants who had heard of the HPV vaccine (see Table 1). Overall, most participants had heard of HPV (62.2%) and the HPV vaccine (61.3%). HPV awareness among non-Hispanic African Americans (59.2%), Hispanics (57.6%), and non-Hispanic Asians (41.8%) were lower compared with HPV awareness among non-Hispanic Whites (66.0%) (p<0.001). Moreover, while 68.0% of non-Hispanic White participants had heard of the HPV vaccine, only 54.5% of non-Hispanic African American participants, 51.1% of Hispanic participants, and 42.5% of non-Hispanic Asian participants reported awareness of the vaccine (p<0.001). 

*HPV Awareness and Associated Factors across Race/Ethnicity*

As shown in Table 2, speaking English “very well” was significantly associated with greater HPV awareness among all participants (OR=2.85, 95% CI=2.25-3.62), as well as in non-Hispanic Whites (OR=2.59, 95% CI=1.68-3.99), non-Hispanic African Americans (OR=5.16, 95% CI=2.42-11.00), and Hispanics (OR=1.61, 95% CI=1.01-2.47). Age and gender were the only two factors that significantly predicted HPV awareness among all participants and the four racial/ethnic groups. While older age was associated with lower HPV awareness, being female was associated with higher HPV awareness. Higher educational attainment was positively associated with HPV awareness among all participants (OR=1.88, 95% CI=1.61-2.20), non-Hispanic Whites (OR=1.96, 95% CI=1.61-2.39), and Hispanics (OR=2.83, 95% CI=1.81-4.43). Additionally, annual household income between $20,000 and $74,000 was significantly related to HPV awareness among all participants (OR=1.46, 95% CI=1.17-1.19), non-Hispanic Whites (OR=1.35, 95% CI=1.00-1.81), and non-Hispanic African Americans (OR=1.86, 95% CI=1.15-3.00). Annual household income over $75,000 was significantly associated with HPV awareness among all participants (OR=1.79, 95% CI=1.39-2.31), non-Hispanic White groups (OR=1.53, 95% CI=1.10-2.14), non-Hispanic African Americans (OR=2.69, 95% CI=1.38-5.22), and non-Hispanic Asians (OR=6.88, 95% CI=1.14-41.63). Although having a regular health care provider was associated with greater HPV awareness among all participants (OR=1.30, 95% CI=1.08-1.57), it was significant only among Hispanics (OR=1.61, 95% CI=1.02-2.53). Increased number of health care provider visits was significantly associated with greater HPV awareness among all participants (OR 1-3 times=1.38, 95%CI=1.10-1.73; OR over 4 times =1.52, 95%CI=1.18-1.97) and non-Hispanic Whites (OR 1-3 times=3.40, 95%CI=1.03-190; OR over 4 times =1.72, 95%CI=1.23-2.41). Having 1 or 2 children in the household was significantly associated with HPV awareness in all participants (OR=1.24, 95%CI=1.00-1.54) and non-Hispanic African Americans (OR=1.81, 95%CI=1.05-3.12). 

*The HPV Vaccine Awareness and Associated Factors Across Race/Ethnicity*

As Table 3 displays, self-report of speaking English very well was found to be significantly related to HPV vaccine awareness in all participants (OR=3.22, 95%CI=2.52-4.12) and in all race/ethnicity groups including Non-Hispanic White (OR=2.19, 95%CI=1.41-3.39), non-Hispanic African American (OR=2.41, 95%CI=1.15-5.01), Hispanic (OR=2.08, 95%CI=1.35-3.19), and non-Hispanic Asian (OR=3.09, 95%CI=1.42-6.73). Age and gender were also associated with HPV vaccine awareness across all participants and all racial/ethnic groups. While older age was associated with lower HPV awareness, female had higher level of HPV awareness. Marital status (OR=1.48, 95%CI=1.20-1.83), having 1 or 2 children (OR=21.44, 95%CI=1.05-1.96), and health status (OR=1.38, 95%CI=1.05-1.82) were only significant predictors of HPV vaccine awareness among non-Hispanic Whites. Education, however, was found to be a significant predictor of HPV vaccine awareness among all participants (OR=1.74, 95%CI=1.48-2.03), as well as non-Hispanic Whites (OR=1.75, 95%CI=1.43-2.14), non-Hispanic African Americans (OR=1.68, 95%CI=1.12-2.52), and HIpanics (OR=2.39, 95%CI=1.57-3.62). household income between $20,000 and $74,999 and household income over $75,000 significantly predicted HPV vaccine awareness among all participants (OR $20,000 and $74,999=1.57, 95%CI=1.24-1.93; OR >$75,000 =2.14, 95%CI=1.09-1.99), non-Hispanic Whites (OR $20,000 and $74,999=1.81, 95%CI=1.1.13-2.89; OR >$75,000 =2.61, 95%CI=1.37-5.00), and non-Hispanic African Americans. Having visited health care providers 1-3 times or over 4 times in the past year was associated with greater HPV vaccine awareness in all groups except Hispanic participants.

**Table 1:** Descriptive of Socio-Demographics, Health Care Resources, Caregiving for Children, Number of Children, Health Status, and English Proficiency (N= 5,868^a^)

		N (%)	Heard of HPV Mean (SD)/ n(%)^c^	p-Value^b^	Heard of HPV Vaccine Mean (SD))/ n(%)^c^	p-Value^b^
Age (Mean=55.8, SD=16.40)			52.10 (15.63)	≤0.001***	52.68 (15.67)	≤0.001***
All		5868 (100)	3648 (62.2)		3600 (61.3)	
Gender	Male	2323 (39.6)	1181 (51.2)	≤0.001***	1105 (48.4)	≤0.001***
	Female	3270 (55.7)	2342 (72.0)		2364 (73.1)	
Race/Ethnicity	Non-Hispanic White	3851 (65.6)	2524 (66.0)	≤0.001***	2578(68.0)	≤0.001***
	Non-Hispanic Black or African American	853 (14.5)	499 (59.2)		456 (54.5)	
	Hispanic	888 (15.1)	510 (57.6)		450 (51.1)	
	Non-Hispanic Asian	276 (4.7)	115 (41.8)		116 (42.5)	
Marital Status	Never married or other	2636 (44.9)	1572 (60.0)	≤0.001***	1460 (56.3)	≤0.001***
	Married or living as married	3182 (54.2)	2051 (64.8)		2112 (67.2)	
Education	Bachelor’s degree	3154 (53.7)	3668 (53.3)	≤0.001***	1644 (53.0)	≤0.001***
	Bachelor’s degree	2678 (45.6)	1966 (73.7)		1938 (73.2)	
Household Income	$0-$19,999	927 (15.8)	445 (48.5)	≤.001***	411 (45.2)	≤0.001***
	$20,000-$74,999	2426 (41.3)	1452 (60.2)		1431 (59.7)	
	$75,000 or more	2024 (34.5)	1486 (73.6)		1497 (74.6)	
Regular Health Care Provider	No	1609 (27.4)	950 (59.4)	≤0.01**	918 (57.8)	≤0.001***
	Yes	4200 (71.6)	2667 (63.9)		2647 (64.0)	
Frequency visiting Health Care Providers	0	838 (14.3)	438 (52.6)	≤0.001***	400 (48.5)	≤.001***
	1-3times	2943 (50.2)	1881 (64.3)		1848 (63.8)	
	4 times and above	2032 (34.6)	1296 (64.2)		1322 (65.8)	
Health Insurance	No	234 (4.0)	132 (56.4)	≤0.05*	124 (53.7)	≤0.001***
	Yes	5120 (87.3)	3024 (64.6)		2980 964.1)	
Caregiver of Children	Yes	367 (6.3)	277 (75.5)	≤.001***	277 (75.9)	≤0.001***
	No	5389 (91.8)	3320 (62.0)		3271 (61.6)	
Number of Children in Household	0	4198 (71.5)	2482 (59.6)	≤0.001***	2460 (59.5)	≤0.001***
	1-2	1145 (19.5)	865 (75.7)		838 (73.7)	
	3 and above	272 (4.6)	192 (70.8)		187 (69.5)	
Health Status	poor/fair	928 (15.8)	496 (54.0)	≤.001***	449 (49.3)	≤0.001***
	Good//very good/excellent	4898 (83.5)	3134 (64.3)		3130 (64.8)	
Speak English	Well/not well/not at all	623 (10.6)	232 (37.4)	≤0.001***	215 (35.2)	≤0.001***
	Very well	5160 (87.9)	3364 (65.6)		3330 (65.4)	

^a^, The total sample size of the study may not be the same as the total sample size of the survey due to missing values. The total sample size of each variable may not be the same as the total sample size of the study due to missing values; ^b^, T-test for continuous variables, Chi-square for categorical variables; ^c^, Mean (SD) for continuous variables, n (%) for categorical variables.

**Table 2:** Regression on Socio-Demographics, Health Care Resources, Caregiving for Children, Number of Children, Health Status, and English Proficiency Predicting HPV Awareness across Race/Ethnicity^a^

Variables	All OR (95% CI)	Non-Hispanic White OR (95% CI)	Non-Hispanic African American OR (95% CI)	Hispanic OR (95% CI)	Non-Hispanic Asian OR (95% CI)
Age	0.961*** (.956, .967)	0.957*** (.950, .964)	0.967*** (.952, .983)	0.957*** (.943, 0.971)	0.968** (0.946, 0.991)
Gender (Ref = Male)					
Female	3.099*** (2.664, 3.606)	3.403*** (2.817, 4.112)	2.256*** (1.464, 3.477)	3.267*** (2.170, 4.919)	2.205* (1.105, 4.399)
Marital Status (Ref = Never married or other)					
Married or living as married	1.077 (0.914, 1.270)	1.123 (0.913, 1.381)	0.974 (0.615, 1.542)	1.175 (0.755, 1.829)	0.613 (0.281, 1.337)
Education (Ref = Bachelor’s degree)					
>=bachelor’s degree	1.883*** (1.609, 2.203)	1.960*** (1.609, 2.388)	1.46 (0.960, 2.221)	2.834*** (1.814, 4.429)	1.816 (0.808, 4.083)
Household Income (Ref=$0 – $19,999)					
$20,000-$74,999	1.459*** (1.172,1.816)	1.348* (1.004, 1.810)	1.858* (1.149, 3.003)	1.076 (0.624, 1.856)	4.369 (0.790, 24.170)
$75,000 or more	1.791*** (1.388, 2.312)	1.533* (1.098, 2.141)	2.685** (1.382, 5.217)	1.431 (0.742,5.757)	6.884* (1.138, 41.629)
Regular Health Care Provider (Ref = No)					
Yes	1.302** (1.080, 1.569)	1.255 (0.975, 1.616)	0.846 (0.535, 1.337)	1.609* (1.023, 2.529)	2.214 (0.969, 5.056)
Frequency visiting Health Care Providers (Ref = 0 time)					
1-3 times	1.376** (1.097, 1.726)	1.397* (1.028, 1.898)	1.551 (0.849, 2.835)	1.493 (0.901, 2.474)	0.704 (0.274, 1.806)
4 times and above	1.526*** (1.184, 1.968)	1.724*** (1.233, 2.411)	1.467 (0.748, 2.877)	1.033 (0.550, 1.939)	1.069 (0.336, 3.404)
Health insurance (Ref=No)					
Yes	1.298 (0.930, 1.811)	1.362 (0.827, 2.242)	2.218 (0.955, 5.152)	1.141 (0.624, 2.084)	0.621 (0.109, 3.542)
Caregiver of Children (Ref = No)					
Yes	1.123 (0.809, 1.560)	1.136 (0.726, 1.777)	0.538 (0.217, 1.329)	1.401 (0.663, 2.957)	1.679 (0.404, 6.980)
Number of Children in Household (Ref =0)					
1 – 2	1.243* (1.002, 1.541)	1.237 (0.916, 1.670)	1.807* (1.047, 3.117)	1.282 (0.792, 2.074)	1.232 (0.551, 2.755)
3 and above	0.82 (0.571, 1.177)	0.794 (0.469)	0.813 (0.336, 1.971)	0.751 (0.365, 1.545)	5.293 (0.602, 46.533)
Health status (Ref = Poor/fair)					
Good/very good/excellent	1.045 (0.847, 1.289)	1.169 (0.893, 1.532)	0.612 (0.362, 1.035)	0.874 (0.513, 1.490)	1.928 (0.617, 6.028)
Speak English (Ref = well/not well/not at all)					
Very well	2.851*** (2.248, 3.616)	2.588*** (1.677, 3.994)	5.155*** (2.415, 11.002)	1.607* (1.046, 2.468)	1.788 (.841, 3.801)
Number of observations	4087	2752	553	593	189
Pseudo R^2^	0.19	0.19	0.186	0.227	0.262
Hosmer-Lemeshow goodness-of-fit test	0.834	0.737	0.634	0.39	0.388

^a^, Binary logistic regression was conducted.

**Table 3:** Regression on Socio-Demographics, Health Care Resources, Caregiving for Children, Number of Children, Health Status, and English Proficiency Predicting HPV Vaccine Awareness Across Race/Ethnicity^a^

Variables	All OR (95% CI)	Non-Hispanic White OR (95% CI)	Non-Hispanic African American OR (95% CI)	Non-Hispanic Hispanic n OR (95% CI)	Non-Hispanic Asian OR (95% CI)
Age (Ref=18yrs – 34yrs)	0.972*** (0.966, 0.977)	0.962*** (0.955, 0.969)	0.980** (0.965, 0.994)	0.974*** (0.961, 0.988)	0.976* (0.954, 0.988)
Gender (Ref = Male)					
Female	4.041*** (3.463, 4.715)	4.947*** (4.054, 6.036)	2.408*** (1.567, 3.701)	3.916*** (2.624, 5.845)	3.822*** (1.897, 7.697)
Marital Status (Ref = Never married or other)	1.438***	1.479***	1.296	1.461	0.82
Married or living as married	(1.219, 1.696)	(1.197, 1.828)	(0.827, 2.031)	(0.953, 2.239)	(0.381, 1.763)
Education (Ref = Bachelor’s degree)	1.737***	1.747***	1.679*	2.385***	1.045
>=bachelor’s degree	(1.484, 2.034)	(1.426, 2.140)	(1.119, 2.520)	(1.570, 3.624)	(0.464, 2.355)
Household Income (Ref=$0 – $19,999)					
$20,000-$74,999	1.547*** (1.242, 1.927)	1.472* (1.091, 1.986)	1.805* (1.128, 2.889)	1.214 (0.715, 2.062)	1.235 (0.299, 5.095)
$75,000 or more	2.143*** (1.657, 2.771)	1.929*** (1.371, 2.714)	2.612** (1.365, 4.996)	1.834 (0.975, 3.447)	1.533 (0.331, 7.086)
Regular Health Care Provider (Ref =No)	1.138	1.243	.607*	1.27	0.81
Yes	(0.944, 1.372)	(0.960, 1.608)	(0.387, 0.952)	(0.821, 1.965)	(0.355, 1.848)
Frequency visiting Health Care Providers (Ref = 0 time)					
1-3 times	1.584*** (1.262, 1.986)	1.498* (1.099, 2042)	2.459** (1.347, 4.489)	1.275 (0.776, 2.096)	2.609* (1.004, 6.780)
4 times and above	2.100*** (1.626, 2.712)	2.167*** (1.540, 3.049)	2.503** (1.276, 4.910)	1.226 (.658, 2.283)	4.499* (1.365, 14.833)
Health insurance (Ref=No)	1.134	1.338	1.121	1.085	0.549
Yes	(0.809, 1.591)	(0.804, 2.226)	(0.472, 2.661)	(0.598, 1.967)	(0.101, 2.979)
Caregiver of Children (Ref = No)	1.016	1.283	0.475	0.826	2.006
Yes	(0.729, 1.417)	(.807, 2.039)	(.201, 1.123)	(.395, 1.728)	(.471, 8.549)
Number of Children in Household (Ref =0)					
1 – 2	1.290* (1.043, 1.594)	1.435* (1.053, 1.955)	1.419 (0.859, 2.345)	1.336 (0.845, 2.112)	1.765 (0.773, 4.031)
3 and above	1.02 (0.707, 1.472)	0.924 (0.535, 1.596)	2.571 (0.953, 6.938)	0.972 (0.484, 1.951)	1.135 (0.155, 8.300)
Health status (Ref = Poor/fair)	1.332**	1.378*	1.147	1.233	0.907
Good/very good/excellent	(1.709, 1.643)	(1.046, 1.815)	(0.696, 1.893)	(0.733, 2.074)	(0.310, 2.654)
Speak English (Ref = well/not well/not at all)	3.221***	2.189***	2.405*	2.075***	3.093**
Very well	(2.521, 4.116)	(1.413, 3.390)	(1.154, 5.013)		(1.422, 6.725)
Number of observations	4070	2741	551	590	188
Pseudo R^2^	0.209	0.216	0.178	0.22	0.244
Hosmer-Lemeshow goodness-of-fit test	0.195	0.9	0.753	0.413	0.3

^a^, Binary logistic regression was conducted.

## Discussion

The current study aimed to investigate factors associated with HPV and HPV vaccine awareness, focusing on English proficiency, across racial/ethnic groups in the U.S. The four racial/ethnic groups examined were: non-Hispanic White, non-Hispanic African American, Hispanic, and non-Hispanic Asian. The current study found that both HPV and HPV vaccine awareness among non-Hispanic African Americans, Hispanics, and non-Hispanic Asians were lower compared with non-Hispanic Whites, which is consistent with previous studies (Garcini et al., 2015; Boakye et al., 2017; Ojinnaka et al., 2017). While over two-thirds of non-Hispanic White participants reported having heard of HPV and the HPV vaccine, only around half of non-Hispanic African American and Hispanic participants reported comparable awareness. Non-Hispanic Asians had the lowest awareness of both HPV and the HPV vaccine. 

The current study revealed that higher levels of English proficiency was positively linked to HPV awareness and awareness of the HPV vaccine. English proficiency was significantly associated with HPV awareness in all groups but Asian Americans. While one previous study found that LEP was associated with lower HPV awareness among Chinese Americans (Nguyen et al., 2012), LEP might not pose a barrier for other non-Hispanic Asians (i.e., Korean American, Vietnamese American). Another possible explanation is that the small sample size of Non-Hispanic Asians (n=189) in this study might generate bias. Future research is needed to examine the role of LEP in improving HPV awareness among larger sample sizes of non-Hispanic Asians and relevant subgroups. 

Concerning HPV vaccine awareness, the current study found that high English proficiency was significantly associated with all four racial/ethnic groups. Although previous studies investigating the association between LEP and the HPV vaccine awareness were not found, Lee et al., (2018) determined the relationship between HPV literacy and LEP and indicated that those with high levels of English proficiency had higher HPV literacy levels than those with a low level of English proficiency among Korean women. While the relationship between LEP and the HPV vaccine among Hispanics was not addressed in the previous study, LEP as a barrier to adequate health communication within this population has been documented in previous studies (Riera et al., 2015; Sarkar et al., 2016). Regarding the negative association between LEP and HPV vaccine awareness also determined among non-Hispanic Whites and non-Hispanic African Americans, one possible explanation is that there were immigrants in those two groups too. Although studies among non-Hispanic White immigrants and non-Hispanic African American immigrants were very rare, a previous study suggested that the more understanding about health among these populations may help improve health disparities in these groups due to their continued increase of population size (Lucas et al., 2005). 

In terms of age, the current study reported the consistent result as past studies, suggesting that older age is associated with a lower level of HPV awareness and the HPV vaccine awareness (Daley et al., 2011; Boakye et al., 2017; McBride and Singh, 2018). In line with previous studies, other variables such as gender (Daley et al., 2011; Boakye et al., 2017; McBride and Singh, 2018), education (Gelman et al., 2011; Boakye et al., 2017; McBride and Singh, 2018), household income (Wong and Do, 2012; McBride and Singh, 2018), having a regular health care provider (Lechuga et al., 2016), and children in the household (Gelman et al., 2011; Allen et al., 2012; Blake et al., 2015; Strohl et al., 2015) had positively significant associations with HPV awareness and HPV vaccine awareness.

Interestingly, study results indicated that visiting a healthcare provider either 1-3 times or over 4 times a year was associated with increased HPV vaccine awareness in all groups except Hispanic, yet having a healthcare provider was positively associated with HPV awareness only among Hispanic participants. A plausible explanation for this result is that the language barrier might impede health communication between this population and healthcare provider (Riera et al., 2015; Sarkar et al., 2016), which posed a barrier to be aware of HPV and the HPV vaccine. However, different from visiting healthcare providers multiple times a year, having a regular provider whom the patients feel comfortable to consult with, might be more helpful for Hispanic to facilitate health communication in spite of language barriers. 

Compared to non-Hispanic African Americans who do not have a child in the household, non-Hispanic African Americans having 1 or 2 children were more likely to have heard of HPV. Previous studies have shown a relationship between having children in the household and HPV awareness (Gelman et al., 2011; Strohl et al., 2015). Gelman and colleagues (2011) focused specifically on having daughters in the home and found that 75% of African American participants had heard of HPV. Of those, only 13% with daughters aged 9-18 had their daughters vaccinated with the HPV vaccine, which the present study confirms since findings did not indicate having children in the home to be associated with HPV vaccine awareness among non-Hispanic African Americans. 

The current study findings should be considered in light of some limitations. First, as a randomized, cross-sectional survey, there are always threats to the generalizability of findings to the broader population. Additionally, the small sample size of non-Hispanic Asian poses an additional threat to representativeness. To increase power and best mitigate the effects of small samples of specific races/ethnicities, subgroups of participants (i.e., Vietnamese, Korean, Chinese, Mexican, Guatemalan, etc.), were combined into larger groups based on their overarching ethnicity (i.e., Asian, Hispanic). However, collapsing these unique subgroups may have obscured unique racial/ethnic differences. Findings may also be limited by HINTS’ use of only one self-reported, dichotomized English proficiency question. Additional and more objective measures are needed to better assess participants’ true level of English proficiency. Moreover, the study population was older (Mean=55.8), which likely makes the reported awareness of HPV and the HPV vaccine an underestimation because most participants were older than the HPV-target population. Lastly, with respect to Hispanic and non-Hispanic Asian, we likely have many immigrants or first-generation Americans in the group of over 65 years old, who might play a substantial part. 

Each racial/ethnic group demonstrated barriers regarding awareness of HPV and the HPV vaccine. With language barriers posing a risk to taking preventive health actions, HPV interventions can be improved by using tailoring materials to individuals’ native languages, particularly for Hispanic and Asian Americans, as existing literature indicates that they frequently experience greater disadvantages regarding English proficiency and have greater difficulty finding health care providers who speak their native languages (Jenny et al., 2013; Reimer et al., 2014; Lechuga et al., 2016; Lee et al., 2018). In addition to developing targeted materials, implementing the presence of an interpreter during interventions and healthcare appointments, particularly in settings with larger populations of non-English speaking individuals, has great potential to address patients’ needs and concerns and aid them in gathering the necessary information. More time allotted for this purpose, such as extended appointment times for non-proficient English speakers, would also help ensure patients receive appropriate attention, assist in improving preventive healthcare measures, and ultimately reduce health disparities. Healthcare professionals and providers would likely benefit from greater education and training on the benefits of the HPV vaccine as well as specific associations between HPV-related cancers and their disparate associations with various racial/ethnic groups. This type of enhanced education could lead to increased promotion of screening, patient education, and greater emphasis on HPV vaccination for cancer prevention, particularly among minority populations. 

Future research and outreach should focus on developing interventions targeted to address the needs of various racial/ethnic groups and individuals with a range of English proficiency. This work should include educational efforts to increase awareness of HPV and related diseases and prevention and screening opportunities. It should also emphasize improving minorities’ access to these services, including medical personnel who speak the language of their patient and aware of their patient populations’ specific risks and needs. By combining multi-level efforts and engagement, measurable, achievable actions can be taken to ultimately reduce HPV-related health disparities and improve HPV-related health outcomes for individuals of every race/ethnicity. 
